# A strategy to apply quantitative epistasis analysis on developmental traits

**DOI:** 10.1186/s12863-017-0508-4

**Published:** 2017-05-15

**Authors:** Marta K. Labocha, Wang Yuan, Boanerges Aleman-Meza, Weiwei Zhong

**Affiliations:** 1 Department of BioSciences, Rice University, Houston, TX 77005 USA; 2 Present address: Institute of Environmental Sciences, Jagiellonian University, Krakow, Poland

**Keywords:** Quantitative epistasis analysis, Genetic interactions, Phenotypes, Multicellular, High-throughput

## Abstract

**Background:**

Genetic interactions are keys to understand complex traits and evolution. Epistasis analysis is an effective method to map genetic interactions. Large-scale quantitative epistasis analysis has been well established for single cells. However, there is a substantial lack of such studies in multicellular organisms and their complex phenotypes such as development. Here we present a method to extend quantitative epistasis analysis to developmental traits.

**Methods:**

In the nematode *Caenorhabditis elegans*, we applied RNA interference on mutants to inactivate two genes, used an imaging system to quantitatively measure phenotypes, and developed a set of statistical methods to extract genetic interactions from phenotypic measurement.

**Results:**

Using two different *C. elegans* developmental phenotypes, body length and sex ratio, as examples, we showed that this method could accommodate various metazoan phenotypes with performances comparable to those methods in single cell growth studies. Comparing with qualitative observations, this method of quantitative epistasis enabled detection of new interactions involving subtle phenotypes. For example, several sex-ratio genes were found to interact with *brc-1* and *brd-1*, the orthologs of the human breast cancer genes *BRCA1* and *BARD1*, respectively. We confirmed the *brc-1* interactions with the following genes in DNA damage response: *C34F6.1*, *him-3* (ortholog of *HORMAD1*, *HORMAD2*), *sdc-1*, and *set-2* (ortholog of *SETD1A*, *SETD1B*, *KMT2C*, *KMT2D*), validating the effectiveness of our method in detecting genetic interactions.

**Conclusions:**

We developed a reliable, high-throughput method for quantitative epistasis analysis of developmental phenotypes.

**Electronic supplementary material:**

The online version of this article (doi:10.1186/s12863-017-0508-4) contains supplementary material, which is available to authorized users.

## Background

A genetic interaction occurs when two mutations at different loci generate a phenotype that cannot be explained by the additive effect of the two single mutations [1]. Genetic interactions are defined as positive (alleviating) when the combination of mutations shows a phenotype that is milder than the expected additive effect from the two single mutations; and negative (aggravating) when the combined phenotype is more severe than expected [1, 2]. Negative interactions can result from the loss of compensatory pathways. Positive interactions may indicate that genes function within a common pathway. Genetic interactions play an important role in many evolutionary processes, such as evolution of sex and recombination [3, 4], robustness and canalization [5], genetic polymorphism [6], and speciation [7]. Genetic interactions are also keys to the complex human diseases [8].

Historically large-scale mapping of genetic interactions mainly focused on synthetic lethality [9–11]. Synthetic lethality occurs when mutations of two genes cause lethality yet mutation of either gene does not [8]. Synthetic lethality is only one type of negative genetic interaction. Synthetic lethality screens would miss non-lethal negative genetic interactions and all positive interactions.

Subsequently, large-scale quantitative epistasis analysis was developed to map the whole spectrum of genetic interactions [12]. In this method, phenotypes are quantitatively measured for double and single mutants. Expected double mutant phenotype is calculated from the single mutant phenotypes using one of the neutrality models [1]. The observed and expected double mutant phenotypes were compared to detect significant differences that indicate genetic interactions. Such method has been applied successfully in unicellular organisms such as yeast and *Escherichia coli* [13–15]. It has also been extended to study metazoan cell lines [16–19].

The next logical step is to extend large-scale quantitative epistasis analysis to intact multicellular organisms. Multicellular organisms have a variety of complex traits. To map the genetic interaction networks underlying those traits, several challenges must be resolved: 1) high-throughput inactivation of two genes in whole multicellular organisms; 2) rapid and quantitative scoring of various phenotypes of interest; and 3) statistical analysis to detect genetic interactions from different phenotypic data.

Here we present a method to resolve those challenges in the nematode *Caenorhabditis elegans*. We applied this method to map the genetic interaction networks regulating two different developmental phenotypes, body length and sex ratio. We validated our interaction results on *brc-1*, a worm ortholog of the human breast cancer gene *BRCA1*, and revealed new genes that regulate DNA damage response and interact with *brc-1*.

## Results

### Collection of quantitative phenotypic data at a large scale

We chose two developmental phenotypes, *C. elegans* sex ratio and body length, to apply quantitative epistasis analysis for the following reasons. First, instead of lethality or general sickness that may involve many biological processes, these are specific developmental traits. Second, these traits represent different types of phenotypic data: the sex ratio measures a population of a binary output from each animal (hermaphrodite/male); the body length measures an individual animal of a continuous variable. A valid quantitative epistasis method should be applicable to both data types and thus be adapted to a wide range of metazoan phenotypes. Third, these phenotypes are easy to score and have biological importance.

**Fig. 1:**
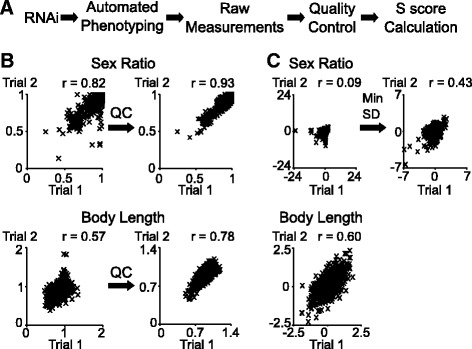
High-throughput method of acquiring quantitative epistasis data. **a** Flow chart showing the experimental process. **b** Reproducibility of raw measurements before (*left panels*) and after (*right panels*) quality control steps. **c** Reproducibility of S scores after quality control steps (*left panels*) and after applying a minimum standard deviation (SD) bound for the sex ratio phenotype (*right panel*). r, correlation; *p* < 0.001 for all r

A research pipeline was developed to enable large-scale quantitative epistasis in *C. elegans* (Fig. 1a). As it is time consuming to generate double mutants, we used RNA interference (RNAi) by feeding [20] on mutant worm background to inactivate the functions of two genes at a high throughput. To enable quantitative phenotyping at a high throughput, we developed an automated imagining system [21, 22] to measure various phenotypes. Sex ratio was measured as percentage of hermaphrodites on a plate. Body length was measured for each worm in μm.

We selected genes whose RNAi caused phenotypes in sex ratio or body length but not general lethality or sickness. For these genes, we obtained homozygous viable mutants that are publicly available. This resulted in 114 RNAi clones and 36 mutants for sex ratio, and 109 RNAi and 31 mutants for body length. Applying RNAi on mutants, 4104 gene pairs were tested for sex ratio, and 3379 gene pairs were tested for body length. For each gene pair, phenotypes of animals with two and one gene inactivated were measured. For each genotype, duplicates or more repeats were tested to measure over 100 animals for sex ratio, and over 40 animals for body length, to enable statistical analysis.

### Data quality control

Quality control (QC) steps are critical for high-throughput large-scale experiments. We first flagged plates with too few worms for manual inspection. While some of these were valid data points indicating synthetic lethality, others were caused by issues such as unfocused images or overcrowded plates, and were thus invalidated.

Next we examined the variation of measurements of the same genotype among replicates tested in the same trial, as well as variation among different trials. A high variation would be flagged for re-testing. Often, one data point deviated from the majority and could not be reproduced in subsequent experiments. We removed such data points as they were likely caused by mistakes such as picking a wrong RNAi bacterial colony.

These QC steps improved data quality. Over 1000 gene pairs for each trait were tested in multiple independent trials, providing a good resource to evaluate the reproducibility of the phenotypic measurements. We split these animals into two groups of similar numbers of worms, and compared these two independent data sets for their consistency. For both sex ratio and body length, the QC steps increased the reproducibility of phenotypic measurements (Fig. 1b), demonstrating improved data quality.

### Normalization of phenotypic measurements

In quantitative epistasis analysis, let *f*_*ab*_, *f*_*a*_, *f*_*b*_ denote the survival rate (fitness) for the animals with two and one gene inactivated respectively, it is expected that *f*_*ab*_ = *f*_*a*_ *f*_*b*_ if the two genes do not interact [23]. Let *p*_*ab*_, *p*_*a*_, *p*_*b*_ denote the lethality rate (phenotypic severity) for the animals with two and one gene inactivated, then *p*_*ab*_ = *p*_*a*_ + *p*_*b*_ − *p*_*a*_ *p*_*b*_ if the two genes do not interact. When *p*_*a*_ and *p*_*b*_ are low, for example, less than 0.1, then *p*_*ab*_ calculation can be approximated to *p*_*ab*_ = *p*_*a*_ + *p*_*b*_ − *p*_*a*_ *p*_*b*_ ≈ *p*_*a*_ + *p*_*b*_. Therefore, either a multiplicative model or an additive model should be used depending on whether the fitness or the phenotypic severity was measured.

To determine whether our phenotypic measurements were fitness or phenotypic severity values, we reasoned that most mutations would reduce the fitness. In comparison with wild-type animals, most sex ratio mutants had reduced hermaphrodite rates; most body length mutants had reduced length. Therefore, we used hermaphrodite rates and length as fitness values. The mutant values were divided by wild-type values to obtain normalized fitness values. The normalized values were then used in the multiplicative model to compute the expected RNAi-on-mutant fitness values from single mutant and single RNAi values.

### Calculation of genetic interaction scores

The next step in quantitative epistasis is using the phenotypic measurements to compute an interaction score. In general, a score of zero indicates no interactions. A score of negative values indicates negative interactions where the phenotype of RNAi-on-mutant animals is more severe than the additive effect of RNAi and mutation. A score of positive values indicates positive interactions where the phenotype of RNAi-on-mutant animals is milder than the additive effect of two single gene inactivation.

One challenge in extending quantitative epistasis analysis to metazoan is to develop interaction scores that can accommodate diverse types of phenotypic data. For example, the scoring method must be adaptable to data with different sample sizes. Our sex ratio data had a small sample size, with most genotypes having fewer than 10 plates tested. In contrast, the body length data had a big sample size, where the median sample size was over 100 worms for any genotype. In previous yeast studies, the median sample size was six colonies for any genotype; accordingly, t-score statistics was used to evaluate interactions because of the small sample size [12]. This would not be appropriate for the body length data with large sample size.

We adapted an S score to detect genetic interactions. The S score is defined as $S=\frac{v_{obs}-{v}_{exp}}{\sigma}$, where *v*_*obs*_ is the observed phenotype of RNAi-on-mutant animals, *v*_*exp*_ is the expected phenotype of these animals if there is no genetic interactions, and σ is the standard deviation of the numerator. The S score is based on z-score statistics, and can thus readily accommodate data with a large sample size. To make it also applicable for data with small sample sizes, we placed a minimum bound [12] for the population standard deviation σ. If an unusually small standard deviation was calculated from the few plates, the minimum bound value was used instead of the calculated standard deviation for σ. This strategy improved the reproducibility of S scores for sex ratio data (Fig. 1c). For data with large sample size such as the body length data, S scores were directly computed without such estimation (Fig. 1c). Using this approach, the reproducibility of our S scores for both sex ratio and body length (correlation of 0.43, and 0.6, respectively, Fig. 1c) was comparable to previous yeast studies (correlation of 0.5) [12].

### Comparison of different genetic interaction scoring methods

**Fig. 2:**
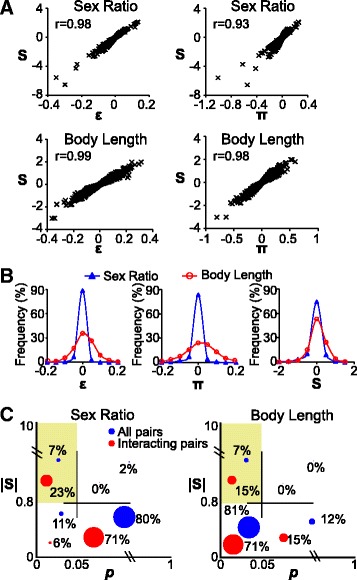
Comparison of different scoring methods for genetic interactions. **a** Correlation (r) between different genetic interaction scores, *p* < 0.001 for all r. **b** Histogram showing the distribution of ε, π, and S scores for sex ratio and body length phenotypes. **c** Enrichment of known interacting pairs over all gene pairs with high |S| scores and low *p* values

Three different interaction scores have been used in previous large-scale quantitative epistasis studies. In addition to the S score, the ε score [12] is defined as ε = *v*_*obs*_ − *v*_*exp*_; the π score [15] is defined as $\pi ={log}_2\left(\frac{v_{obs}}{v_{exp}}\right)$. All three scores were highly correlated for both sex ratio and body length data (Fig. 2a), suggesting that they were in principle detecting the same interactions.

A key difference among the three scores was that S scores took variation into consideration, whereas the other two scores relied solely on mean values. As different traits have different variations, adjustment with variations enabled S scores to be comparable across different traits. For example, body length is more variable than sex ratio in *C. elegans*. Accordingly, the distribution of ε scores and π scores was more widely spread in body length than in sex ratio (Fig. 2b). In contrast, the distribution of S scores of the two traits appeared similar (Fig. 2b). Therefore, S scores are more versatile than the other scoring methods as it can provide a consistent scoring scale for different traits.

### Comparison of different thresholds

Several thresholding methods can be applied to interaction scores to determine which genes interact. First, an empirically-determined cutoff can be directly applied to the values of S scores [18]. Second, assuming that S scores follow a normal distribution, Z scores of these S values can be used as a threshold. Thresholds for Z scores are commonly set to Z > 1.96 or Z > 4 [15, 24], corresponding to *p* < 0.05 and *p* < 0.0001, respectively. Third, if there are multiple independent trials of S score testing for each gene pair, then these S scores from different trials can be compared with zero using statistical tests such as t-test to calculate *p*-values of this gene pair having a non-zero S score. As such test is performed for each gene pair, *p*-values are usually adjusted for false discovery rate (FDR). Then adjusted *p*-value (usually *p* < 0.05) can be used to determine which scores are considered significant [19].

We tested the first method of thresholding S scores. The uniformity of S scores across different traits enabled us to use the same threshold value of S for both sex ratio and body length data. To determine a proper threshold, we queried WormBase for known genetic interactions, and found 31 sex ratio interactions and 41 body length interactions. Guided by these known interactions, we empirically determined the absolute value of S (|S|) over 0.8 as the threshold for interaction. Known interacting pairs were enriched with |S| values over 0.8: a 2.6-fold enrichment (23% vs. 9%) was observed for sex ratio and a 2.1-fold enrichment (15% vs. 7%) was observed for body length (Fig. 2c), suggesting that this |S| threshold can effectively capture interacting genes.

We also examined the second method of using Z scores of S scores as thresholds. Z scores of 1.96 corresponded in our study to S scores of 0.75 and −0.83 for sex ratio and 0.46 and −0.75 for body length (Additional file 1: Table S1). Therefore, the first method of |S| > 0.8 is in general more stringent than this method Z > 1.96.

Finally, we compared the performance of |S| and *p* values. As we did not have multiple biological replicates for each gene pair, we could not calculate *p*-values comparing S scores from multiple trials with the value of 0. Instead, we calculated *p*-values comparing the expected and observed fitness values of RNAi-on-mutant animals. Because sex ratio is based on counts of two types of animals (hermaphrodites/males) and body length is a continuous measurement, different statistical tests were required to analyze these data. Exact binomial test was used on sex ratio data to compare the observed sex ratio of RNAi-on-mutant animals with the expected one, whereas Student t-test was used on body length data to compare the observed body length of RNAi-on-mutant animals with the expected one [25]. Calculated *p*-values were then adjusted for multiple comparisons [26]. We found that known interacting pairs were mildly enriched with *p* < 0.05 for sex ratio with a 1.6-fold enrichment (29% vs. 18%), but not enriched for body length (86% vs. 88%, Fig. 2c). Therefore, *p* value alone was not a strong indicator of interactions. Combining both *p* values and |S| scores did not improve performance for the body-length data, but provided a slightly better performance than |S| alone for sex-ratio data: the enrichment was 3.3 fold (23% vs. 7%) among interacting genes in comparison with the 2.6 fold using |S| alone (Fig. 2c). Overall, these data suggested that those *p* values had limited effects on distinguishing interacting gene pairs in our data.

All these data suggested that the first threshold of |S| > 0.8 was the most conservative cutoff for genetic interactions, and that it was also most consistent in both traits. In addition, this threshold required no statistical assumptions. Considering these factors, we concluded that using the S score values |S| > 0.8 as a cutoff was the most effective thresholding method for interactions.

### Discovery of new genetic interactions

Using |S| > 0.8 and *p* < 0.05 as the cutoffs, our analysis revealed 288 sex-ratio genetic interactions, including 132 synthetic lethality, and 228 body-length genetic interactions (Additional file 1: Table S1). 98% (504/516) of these interactions have not been reported previously, suggesting that our previous knowledge on genetic interactions was incomplete and that quantitative analysis could improve our understanding on genetic interactions.

**Fig. 3:**
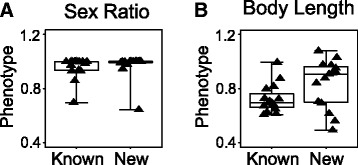
Quantitative epistasis can detect interactions for mutants with subtle phenotypes. **a** Sex ratio phenotypes. **b** Body length phenotypes. Box plots show phenotype distribution for mutants with previously known genetic interactions (Known) and for mutants with only interactions discovered in this quantitative study (New). Mutant phenotypes were divided by wild-type values to be normalized, so that 1 indicates wild-type

Comparing these newly discovered interactions with previously known interactions, we noted that previously known interactions tend to involve genes with more severe phenotypes (Fig. 3a and b). Among the sex ratio mutants that were associated with previously known interactions, the median hermaphrodite rate was 95.5% of the wild-type value (Fig. 3a). Among the group of mutants without known interactions, many of them were close to wild-type, with the median hermaphrodite rate being 99.8% of the wild-type value (Fig. 3a). A similar trend was observed for the body length data: mutants with known interactions had more severe phenotypes (median being 69.5% of the wild-type value) than mutants without known interactions (median being 90.7% of the wild-type value) (Fig. 3b). As previously known interactions were detected using qualitative observations, these data suggested that quantitative epistasis analysis was particularly effective with genes of subtle phenotypes.

### Computational validation of genetic interactions

Standard criteria were applied to validate our methodology. First, the method must generate reproducible data. We confirmed that our S scores were reproducible with a correlation between trials similar to that in yeast studies (Fig. 1c). Second, genetic interactions are expected to be sparse. We confirmed that the distributions of our S scores were centered at zero (Fig. 2b), and that only 6.9% (516/7483) of gene pairs were interacting. Such rate of interactions was similar to that in studies of *E. coli* (7%) [24] and *S. pombe* (6%) [14]. Finally, the method must recapture known interactions. We confirmed that known interacting pairs were enriched with high |S| scores (Fig. 2c). These data validated our methodology in detecting genetic interactions.

It is expected that interacting genes often share similar functions. We thus evaluated whether the interactions we discovered were consistent with known functional annotations. We partitioned the genetic interaction networks using the METIS software [27]. Three groups of densely connected genes were identified in sex ratio and body length networks (Additional file 2: Table S2). Many genes partitioned into the same groups shared similar functions, demonstrating the validity of the interactions. For example, among sex ratio genes, six dosage compensation genes [28], *sdc-1*, *sdc-2*, *dpy-21*, *dpy-26*, *dpy-27*, and *dpy-28*, were partitioned into the same group (Group 2, Additional file 2: Table S2). Another group (Group 3, Additional file 2: Table S2) contained seven genes involved in DNA damage response (GeneOntology.org), *brc-1*, *brd-1*, *cep-1*, *F26B1.2*, *mre-11*, *rad-51*, and *rfs-*1. Among them, *brc-1*, *brd-1*, *cep-1* are known to interact with each other [29]. The consistency in biological functions suggested that our method is detecting true genetic interactions.

### Discovery of new *brc-1* interactions

One of the sex ratio genes we examined was *brc-1*, the ortholog of the human gene *BRCA1*. In humans, *BRCA1* was associated with early onset of breast and ovarian cancer [30]. *BRCA1* physically binds to *BARD1* [31], another protein that was linked to breast cancer susceptibility [32]. *BRCA1* functionally interacts with *RAD51* in DNA damage repair [33]. In worms, mutants of *brc-1* and the *BARD1* ortholog *brd-1* had elevated numbers of apoptotic germ cells before and after irradiation, and increased embryonic lethality after irradiation [29], suggesting that they function in DNA damage repair.

**Fig. 4:**
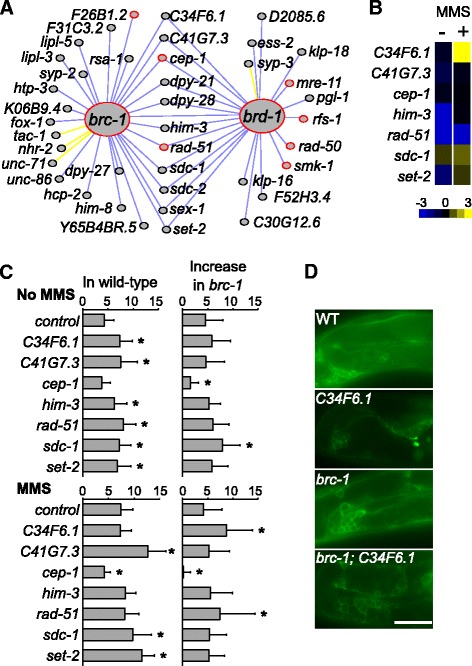
*brc-1* interactions. **a** Genes interacting with *brc-1* and *brd-*1 in sex ratio. *Red circles* indicate genes associated with DNA repair in Gene Ontology; *Blue lines* indicate negative interactions (S < −0.8); *Yellow lines* indicate positive interactions (S > 0.8). **b** *brc-1* genetic interactions in embryonic survival with (+) and without (−) MMS exposure. Heat map displays S scores. *Blue*, S < 0; *Black*, S = 0; *Yellow*, S > 0. **c** *brc-1* genetic interactions in apoptotic germ cell numbers with or without MMS exposure. *Left panels* display the number of apoptotic cells of various RNAi on wild-type background. *Right panels* display the increase of apoptotic cells in *brc-1* mutant background. *Bars* and *error bars* indicate mean and standard deviation. *, *p* < 0.05 in comparison with the control RNAi group. **d** Images of apoptotic germ cells showing that *brc-1* mutation interacts synergistically with RNAi of *C34F6.1*. WT, wild-type. Scale bar, 10 μm

Our quantitative epistasis analysis on sex ratio revealed 38 genes interacting with *brc-1* or *brd-1* (Fig. 4a). Seven of them, *cep-1*, *F26B1.2*, *mre-11*, *rad-50*, *rad-51*, *rfs-*1, and *smk-1*, are involved in DNA repair (GeneOntology.org). We recaptured known *brc-1* interactions with *brd-1* and the *P53* ortholog *cep-1* [29]. The *RAD51* ortholog *rad-51* was also found to interact with *brc-1*. These results demonstrated the effectiveness of our method in detecting genetic interaction.

In addition to recapturing known interactions, our analysis on sex ratio also revealed new *brc-1* interactions. For example, 11 genes, *C34F6.1, C41G7.3, cep-1, dyp-21, dpy-28, him-3, rad-51, sdc-1*, *sdc-2*, *set-2*, and *sex-1*, were found to interact with both *brc-1* and *brd-1* (Fig. 4a). Among them, only *cep-1* was known to interact with *brc-1* in worms. To validate the new interactions, we tested these genes on a different phenotype, DNA damage response. We reasoned that if these genes interact with *brc-1*, then they are also likely to be involved in DNA damage response. To evaluate DNA damage response, we measured embryonic lethality and apoptotic germ cells with and without exposure to the DNA damaging reagent methyl methanesulfonate (MMS). Four genes were not included in the test for technical reasons: *sdc-2* and *sex-1* were not included because they had synthetic lethality with the *brc-1* mutant; *dyp-21* and *dpy-28* were not included because these worms had egg laying defects that caused difficulties in scoring embryonic lethality, and their squeezed organs in the dumpy body caused difficulties in scoring apoptotic germ cells.

We applied RNAi of seven genes, *C34F6.1, C41G7.3, cep-1, him-3, rad-51, sdc-1*, and *set- 2*, on the *brc-1* mutant, and scored the embryonic survival rate with and without MMS exposure. The interaction score S was calculated, and the threshold |S| > 0.8 and *p* < 0.05 was applied. Five out of the seven genes, *C34F6.* *1*, *him-3*, *rad-51*, *sdc-1* and *set-2*, were found to interact with *brc-1* before or after MMS exposure (Fig. 4b, Additional file 3: Table S3).

RNAi of these five genes also caused a significant increase of apoptotic germ cells (Fig. 4c, Additional file 3: Table S3), confirming the function of these genes in DNA damage response. Our experiment also confirmed previously reported [29] interaction between *brc-1* and *cep-1* in regulating apoptotic cells (Fig. 4c). Interestingly, while *brc-1* mutation caused a 4.5-cell increase of apoptotic germ cells on wild-type background (4.3 ± 1.9 vs. 8.8 ± 3.5), the same mutation caused an 8-cell increase in the *sdc-1(RNAi)* background (7.2 ± 2.3 vs. 15.2 ± 3.5) (Fig. 4c, Additional file 3: Table S3), suggesting a synergistic effect between *sdc-1* and *brc-1* inactivation in generating apoptotic cells. Similar effects with *brc-1* were also observed for *rad-51* and *C34F6.1* RNAi after MMS exposure (Fig. 4c, d, Additional file 3: Table S3). Together, these data confirmed the *brc-1* interactions with *C34F6.1*, *rad-51*, and *sdc-1*.

Overall, we tested six new *brc-1* interactions, *C34F6.1, C41G7.3, him-3, rad-51, sdc-1*, and *set-2*, for their functions in DNA damage response. Five of them, *C34F6.1*, *him-3*, *rad-51*, *sdc-1* and *set-2*, were found to interact with *brc-1* in embryonic survival before or after MMS exposure. RNAi of all genes caused an increase of apoptotic germ cells. *C34F6.1*, *rad-51*, and *sdc-1* were also found to interact with *brc-1* in apoptotic germ cells before or after MMS exposure. While *C34F6.1* and *sdc-1* do not have a clear human ortholog, *rad-51* is orthologous to human *RAD51*, *him-3* is orthologous to human *HORMAD1* and *HORMAD2*; *set-2* is orthologous to human *SETD1A*, *SETD1B*, *KMT2C*, and *KMT2D*. One of those interactions, with *rad-51*, is known to be conserved in humans [34, 35]. Similar to the *brc-1* ortholog *BRCA1*, *HORMAD*1 [36], *SETD1A* [37], *KMT2C* [38, 39], and *KM2D/MML4* [40, 41] have all been linked to breast cancer, suggesting that these genetic interactions may be conserved in human cells.

## Discussion

We describe here a methodology to extend quantitative epistasis analysis to metazoan. We validated our results both computationally and experimentally. Computationally, we evaluated the reproducibility of S scores and the consistency with known interactions. Experimentally, we tested the *brc-1* sex-ratio interactions with an orthogonal phenotype: DNA damage response. Our results demonstrated that the principles of quantitative epistasis analysis remain the same from single cells to whole animals. While *C. elegans* has some unique advantages such as feeding RNAi, genome editing tools enable inactivation of two genes in many species. Therefore, it is possible to extend this quantitative epistasis analysis to other phenotypes in other multicellular organisms.

## Conclusions

We have developed and validated a method for quantitative epistasis analysis on different developmental traits. Application of the method on *C. elegans* sex ratio and body length has enabled us to detect over 500 new genetic interactions including genes interacting with the breast cancer gene ortholog *brc-1*. This method can be used to study genetic interactions of phenotypes in *C. elegans* and it provides guidelines for developing similar methods in other metazoans.

## Methods

### Gene selection

Genes were selected based on WormBase (version 225) RNAi phenotype annotation of *high incidence of males* (*him)* for sex ratio phenotype, and reported RNAi phenotype of *dumpy*, *small* or *long* for body length [42]. RNAi bacterial clones were taken from the Ahringer RNAi library [42], or the ORFeome-RNAi library [43]. HT115 bacteria with the empty RNAi vector L4440 was used as a negative control.

Homozygous viable mutants were obtained from the *Caenorhabditis* Genetics Center (CGC). Unoutcrossed strains were outcrossed six times (all WWZ strains). Bristol N2 strain was used as wild-type. The following 36 sex-ratio mutants were used: CB1256 *him-3(e1256)* IV, CB1416 *unc-86(e1416)* III, CB1489 *him-8(e1489)* IV, CB4088 *him-5(e1490)* V, CB428 *dpy-21(e428)* V, CB5380 *fox-1(e2643)* X, CB541 *unc-71(e541)* III, CV138 *sgo-1(tm2443)* IV, DW102 *brc-1(tm1145)* III, DW103 *brd-1(dw1)* III, JK3101 *fbf-2(q738)* II, MH801 *sur-7(ku119)* X, MT1080 *sdc-1(n485)* X, MT1446 *her-1(n695)* V, MT14851 *set-2(n4589)* III, MT2244 *sel-10(n1077)* V, SP488 *smk-1(mn156)* V, WWZ238 *htp-3(gk26)* I, WWZ239 *gpr-2(ok1179)* III, WWZ240 *rfs-1(ok1372)* III, WWZ241 *klp-10(ok704)* IV, WWZ242 *cki-2(ok2105)* II, WWZ243 *skr-1(ok1696)* I, WWZ244 *atl-1(ok1063)* V, WWZ245 *hil-4(ok1945)* V, WWZ246 *C30G12.6(ok2389)* II, WWZ247 *ess-2(ok3569)* III, WWZ249 *pme-3(gk120)* IV, WWZ250 *F52H3.4(ok2692)* II, WWZ251 *T08D2.7(ok431)* X, WWZ252 *zhp-3(ok1993)* I, WWZ255 *hcp-2(ok1757)* V, WWZ256 *coh-3(gk112)* V, WWZ257 *lipl-5(ok3581)* V, WWZ258 *W02D9.3(ok2857)* I, XY1054 *cep-1(lg12501)* I. The following 31 body-length mutants were used: BE93 *dpy-2(e8)* II, CB1166 *dpy-4(e1166)* IV, CB12 *dpy-9(e12)* IV, CB128 *dpy-10(e128)* II, CB130 *dpy-8(e130)* X, CB14 *dpy-6(e14)* X, CB1482 *sma-6(e1482)* II, CB187 *rol-6(e187)* II, CB224 *dpy-11(e224)* V, CB27 *dpy-3(e27)* X, CB491 *sma-3(e491)* III, CB88 *dpy-7(e88)* X, CT11 *hbl-1(mg285)* X, DA2154 *phb-2(ad2154)* II, DR1369 *sma-4(e729)* III, DR933 *dpy-13(m399)* IV, FF41 *unc-116(e2310)* III, FX1053 *ins-11(tm1053)* II, JJ1237 *mex-6(pk440)* II, JK2729 *dpy-18(ok162)* III, JR2370 *egl-18(ok290)* IV, MT5998 *sem-5(n2195)* X, NG41 *sex-1(gm41)* X, RB1093 *C08H9.2(ok1071)* II, TN322 *dpy-20(cn322)* IV, WWZ127 *M195.2(ok1503)* II, WWZ130 *sams-5(gk147)* IV, WWZ131 *fat-3(ok1126)* IV, WWZ136 *F42A8.1(ok2579)* II, WWZ137 *F54C4.3(ok2037)* III, VC2428 *sams-1(ok2946)* X.

Animals were grown at 20 °C on standard nematode growth media (NGM) seeded with the OP50 strain of *Escherichia coli* as described in Stiernagle [44].

### RNAi

RNAi was performed on solid media as described [20]. 6-well NGM plates with 50 μg/ml carbenicillin and 1 mM IPTG were used. Bacteria were cultured overnight at 37 °C in 96-well deep-well plates in L-broth with 50 μg/ml carbenicillin. Seventy microliter of bacteria were seeded onto each well of RNAi plates, and incubated at room temperature overnight.

For sex ratio, depending on the fecundity of each worm strain, 5 to 15 L4 hermaphroditic larvae were picked onto each well of 6-well plates seeded with RNAi bacteria, cultured at 25 °C for 20 to 30 h, and removed, so that each well had ~100 eggs. The eggs were cultured for 2 days at 25 °C so that the animals reached adulthood. The animals were then scored for the sex ratio phenotype.

For body length, worms were synchronized by bleaching gravid adults to obtain eggs and cultured in M9 buffer with 5 μg/ml cholesterol at 20 °C overnight to obtain synchronized L1 larvae [44]. About 100 L1 s were dropped to each well of the 6-well seeded RNAi plates. Animals were grown for 48 to 56 h at 25 °C until animals on control bacteria reached adulthood.

### Automatic phenotyping

For sex ratio, adult animals were washed off from RNAi plates with S Basal solution [44], and transferred to unseeded scanning plates (modified NGM plates that do not contain peptone or cholesterol). The scanning plates were left without lids for about 30 min to air dry. Ten microliter of 1 M sodium azide was then added to each well to kill the animals.

For body length, 20 μl of 1 M sodium azide was added to each well of the RNAi plates to kill the animals. All RNAi on the same strain of worms were killed at the same time, and scanned immediately afterwards.

### Quality control

Several quality control steps were employed to detect potential experimental errors. 1) A well was flagged for manual examination if it had fewer than 10 animals in sex ratio experiments [22] or 20 animals in body length experiments. 2) Wells with standard deviation of body length over 250 μm were re-tested. Empirically, we found these wells often had experimental issues such as worm crowding, or poor focusing during scanning. 3) As we tested at least duplicates for each genotype in each experiment, consistency between the replicates in the same experiment was examined using coefficient of variation (CV) of hermaphrodite percentage or mean length from each well. All genotypes with CV > 15% were re-tested in another experiment. 4) Consistency of the same genotype tested in different experiments was examined using the absolute difference (AD) among measured phenotypes. If the maximum AD was over 12% of wild-type values, then the genotype was re-tested. 5) The minimal number of animals tested for each genotype must be at least 100 for sex ratio and 40 for body length. Additional testing was conducted if the total number of animals were below this threshold. All thresholds were chosen empirically.

### Calculation of genetic interaction scores

For sex ratio, male and hermaphrodite counts from all experiments were summed up to calculate the percentage of hermaphrodites for a given genotype. The percentage of hermaphrodites was then divided by the wild-type value to obtain the normalized fitness measurement. Let *w*_*ab*_, *w*_*a*_, and *w*_*b*_ denote the normalized fitness for RNAi-on-mutant, RNAi, and mutant animals, respectively, then the observed fitness of RNAi-on-mutant animals is *v*_*obs*_ = *w*_*ab*_; the expected fitness of RNAi-on-mutant animals is *v*_*exp*_ = *w*_*a*_ *w*_*b*_. To compute $S=\frac{v_{obs}-{v}_{exp}}{\sigma}$, the standard deviation σ was calculated as $\upsigma =\sqrt{\sigma_{a b}^2+{\sigma}_a^2{w}_b^2+{\sigma}_b^2{w}_a^2}$, where *σ*_*ab*_, *σ*_*a*_, *σ*_*b*_ denote the standard deviation for animals with two and one gene inactivated, respectively. Standard deviation was calculated using the hermaphrodite percentage of each well as a data point, normalizing it using wild-type values, and combining data points from all experiments to compute the standard deviation. If there are fewer than six wells available for *σ*_*ab*_ calculation, then the median standard deviation from all RNAi on this mutant was used for *σ*_*ab*_. The minimal bound for σ was set to 0.04. This threshold was based on the average σ calculated from all genotypes with at least six wells available for *σ*_*ab*_ calculation.

For body length data, the growth time of the same genotype from different experiments could vary up to 4 h, while the growth time for all RNAi on the same worm strain had the exact same growth time in a given experiment. Consequently, we chose not to combine the length measurement directly from different experiments, but instead computed and combined RNAi impact from different experiments. The RNAi impact for mutant *a* on *b* RNAi was ${r}_{a b}=\frac{L_{a b}}{\overline{L_a}}$, where *L*_*ab*_ denote the length of an animal with two genes inactivated, and $\overline{L_a}$ denote the mean length of the single mutant *a* tested in the same experiment. Similarly, let *L*_*b*_ denote the length for a wild-type worm fed with RNAi bacteria *b*, $\overline{L_{wt}}$ denote the mean length of all wild-type worms fed with control bacteria in the same experiment, then the RNAi impact for a wild-type worm on *b* RNAi was ${r}_b=\frac{L_b}{\overline{L_{wt}}}$. The observed and expected fitness values of RNAi-on-mutant animals were calculated as ${v}_{obs}=\overline{r_{ab}}$, ${v}_{exp}=\overline{r_b}$, where $\overline{r_{ab}}$ and $\overline{r_b}$ denote the mean values of *r*_*ab*_ and *r*_*b*_ combined from all experiments. To compute $S=\frac{v_{obs}-{v}_{exp}}{\sigma}$, the standard deviation σ was calculated as $\upsigma =\sqrt{\sigma_{ab}^2+{\sigma}_b^2}$, where *σ*_*ab*_, *σ*_*b*_ denote the standard deviations for *r*_*ab*_ and *r*_*b*_ combined from all experiments.

### DNA damage experiment

The strain ZH231 *unc-76(e911) V; enIs7 [Pced-1::ced-1::GFP + unc-76(+)]* was used as wild-type control. This strain was kindly provided by Dr. Zheng Zhou at Baylor College of Medicine, Houston, TX. In this strain, apoptotic cells were highlighted by green fluorescence, allowing easy visualization of apoptotic cells [45]. This strain was crossed with the strain DW102 to generate WWZ125 *brc-1(tmll45); enls7[pced-1 ced-1::gfp and punc-76(+)]*, which was used as *brc-1* mutant.

L4 worms were fed with different RNAi clones and removed after they reached adulthood and laid eggs. Those eggs were left on RNAi plates to develop to the L4 larval stage and then transferred onto new RNAi plates with or without 0.05 mg/ml methyl methanesulfonate (MMS) added to the agar. After 24 h, 10 to 20 worms were examined under a Zeiss AxioImager M2 m microscope for green fluorescence of apoptotic cells. Another 10 worms were moved to fresh RNAi plates with or without MMS for egg laying. Eggs were transferred to fresh RNAi plates. After 24 h, unhatched eggs were counted to evaluate embryonic lethality. Over 120 embryos were scored for each genotype. Minimum nine worms for each genotype were scored for apoptotic cells. S score calculation for embryonic survival was the same as that for the sex ratio phenotype.

### Statistical analysis

The exact binomial test [25] was used to compare observed and expected values for sex ratio and survival rate of RNAi-on-mutant animals. The student t-test [25] was used on body length data to compare the observed and expected RNAi impact on mutants. Apoptotic cell numbers were compared using the ANOVA test followed by contrast analysis [25]. The *p*-values from all statistical tests were adjusted for multiple comparison (separately for each phenotype) using Benjamini-Hochberg correction with family-wise error set to 5% [26] to control for false discovery rate. All tests were performed in R [46].

## Additional files

Additional file 1: Table S1. Genetic interactions with S scores, Z scores and *p* values. (XLSX 456 kb)

Additional file 2: Table S2. Partition of genetic interaction networks showing functional groups. (XLSX 11 kb)

Additional file 3: Table S3. DNA damage data for *brc-1* interactions. (XLSX 12 kb)

## Abbreviations

AD: Absolute difference
CGC: *Caenorhabditis* Genetics Center
CV: Coefficient of variation
FDR: False discovery rate
*him*: High incidence of males
MMS: Methyl methanesulfonate
NGM: Nematode growth media
QC: Quality control
RNAi: RNA interference
SD: Standard deviation
WT: Wild-type
